# Primary care providers are, fundamentally, risk managers – And this is a challenge for health policy

**DOI:** 10.1016/j.lanwpc.2020.100037

**Published:** 2020-10-02

**Authors:** Benjamin Daniels

**Affiliations:** Georgetown University, Washington, DC, USA

**Keywords:** Primary care, China

There seems to be a consensus that the quality of primary care around the world is unreasonably poor, and study after study is conducted to point this out. Health care providers are performing badly in public systems; they are doing the same in private settings. At the same time, the motivations of individual providers are unquestionably noble, with only have the best health of patients at heart.

If providers have good intentions but perform poorly, they must face either a resource shortage like equipment or education or a constraint like profit motivation or bad management [1]. Yet endless interventions find that external efforts to remove these constraints rarely substantially improve performance [2]. So providers must be intentionally choosing to perform as they do – and this would imply their beliefs about what is best for patients are wrong.

But this does not comport with the evidence. We repeatedly find that patients have access to providers who are reasonably knowledgeable and that those providers exert effort to meet patient needs [3,4]. In vignette studies, we find that providers in resource-poor settings have sufficient (though contextually variable) knowledge to manage common conditions. In standardized patient studies, we find that providers are highly responsive to small changes in presentation, suggesting they work hard to solve each case [5]. We frequently observe an aggressive approach to care covering many potential causes and symptoms, with conscientious cost control for patients [6]. So why, then, does care remain poor?

A growing body of research suggests a fundamental tension in early diagnosis, when primary care providers take wide differential diagnoses, assess likely risks, and decide which are worth acting on [7]. In the course of a short interaction, providers make a management decision balanced between doing *too little* to help the patient and doing *too much*. No provider, presumably, wants to be responsible for ignoring a critical warning sign leading to death; but none wants to be responsible for overusing expensive diagnostics and prescribing dozens of medications “just in case”. For each patient, the provider has to adjudicate what might be called “the WebMD problem”: it's probably nothing, but it *might* be cancer.

How, then, are providers deciding what to do? Two studies in *The Lancet Regional Health – Western Pacific* help us reframe our thinking of how health care providers face these decisions. Yi et al (2020) assess the ability of rural health care providers in Southwestern China to manage epilepsy using clinical vignettes [8]. Most providers say they would do little to “treat” the condition, including even asking relevant history questions. But this does not mean their management of the patient was poor. Fully 90% expressed this was a case requiring specialized attention and said they would refer accordingly. In Guo et al. (2020), clinical vignettes were used to assess ability to diagnose and manage angina [9]. When the providers were told the correct diagnosis, they improved only slightly in correct management – but use of potentially harmful medications dramatically fell.

In both studies, we see providers making behaviors of large magnitudes where the risk of misdiagnosis can be controlled. Epilepsy is easy to diagnose based on hallmark presentations of grand mal seizures. But the risk of a serious neurological problem is hard to assess (particularly in a child, as the study vignette depicts). Chest pain, too, is easy to identify, but again difficult to quickly separate mundane causes from life-threatening risks. Correspondingly, we see that providers don't get much better at treating angina when told what it is – if they worried it was serious, that was already covered (by ordering referral). But they stopped taking additional behaviors potentially intended to manage other causes. Across the research, then, we see a common thread: perfect diagnosis is hard at the primary level, and providers knowingly behave correspondingly to contain catastrophic tail risks.

This creates a policy problem. The motivation for some of this research (including, explicitly, Guo et al) is that some governments now want to conserve resources by having primary care providers manage complex and chronic conditions. But what primary care providers have revealed themselves to be supremely competent at is not diagnosis and treatment, but *risk assessment and triage* – determining whether a serious underlying condition is possible, and if so, referring appropriately. The proliferation of specialized laboratory diagnostics now often makes this an optimal choice for a risk-averse provider whenever they are not completely confident in their diagnosis (and in standardized patients, for example, formal diagnoses are rare).

A policy shift towards primary care providers taking on that risk – by demanding providers refer less, treat more, and manage complex and chronic conditions directly – appears fundamentally at odds with how primary care operates globally today. Therefore, these policy suggestions call for serious academic and policy consideration of whether this is an appropriate model for primary health care and the development of tools to cope with such a fundamental change in approach.

## Declaration of Competing Interest

The author declares that there is no conflict of interest.
